# A Novel Multiband Spectrum Sensing Method Based on Wavelets and the Higuchi Fractal Dimension

**DOI:** 10.3390/s19061322

**Published:** 2019-03-16

**Authors:** Yanqueleth Molina-Tenorio, Alfonso Prieto-Guerrero, Rafael Aguilar-Gonzalez

**Affiliations:** 1 Master of Sciences and Information Technologies, Metropolitan Autonomous University Iztapalapa, Mexico City 09360, Mexico; yanqueleth@xanum.uam.mx; 2 Electrical Engineering Department, Metropolitan Autonomous University Iztapalapa, Mexico City 09360, Mexico; r.aguilar@xanum.uam.mx

**Keywords:** cognitive radios, multiband spectrum sensing, multiresolution analysis, continuous wavelet transform, Higuchi fractal dimension

## Abstract

In this work, two novel methodologies for the multiband spectrum sensing in cognitive radios are implemented. Methods are based on the continuous wavelet transform (CWT) and the multiresolution analysis (MRA) to detect the edges of available holes in the considered wideband spectrum. Besides, MRA is also combined with the Higuchi fractal dimension (a non-linear measure) to establish the decision rule permitting the detection of the absence or presence of one or multiple primary users in the studied wideband spectrum. Methods were tested on simulated and real signals showing a good performance. The results present these two methods as effective options for detecting primary user activity on the multiband spectrum. The first methodology works for 95% of cases, while the second one presents 98% of effectivity under simulated signals of signal-to-noise ratios (SNR) higher than 0 dB.

## 1. Introduction

The cognitive radio (CR) concept is considered as a radio with the capacity to opportunely take advantage of the spectral gaps to continue transmitting [1]. CR has been widely considered in our days as one of the outstanding solutions to spectrum scarcity. CR techniques provide the ability to use or share the spectrum in a timely manner and operate in the best available channel. In this way, CR technology will allow to secondary users (SUs), also called unlicensed users, to determine what parts of the spectrum are available and detect the presence of licensed users or primary users (PUs). When an SU operates in a non-authorized band, the CR selects the best available channel, coordinates its access to this channel and, in the right moment, vacates the channel when a PU is detected [2]. Therefore, the CR paradigm involves the stages of sensing, decision, sharing, and mobility of the spectrum [3]. The first stage of CR, i.e., spectrum sensing (SS), is fundamental to determine the presence of PUs and it is the goal of this work.

Spectrum sensing in a CR is a term which implies the obtaining of the characteristics of the spectrum using multiple dimensions, such as time, space, frequency, and code. Among these characteristics are modulation type, waveform, bandwidth, carrier frequency, etc. In general, the different techniques involved in the SS are focused on detecting PUs in only one band [4]. Future wireless communications services require high throughputs; this can be reached with more bandwidth. However, in several scenarios, available spectrum spaces are in non-contiguous frequencies. Due to this, it is necessary to have a wide panorama of PU activity. A solution for that is considering multiband spectrum sensing, a branch of wideband spectrum sensing [4].

A first introduction to the concept of multiband spectrum sensing appears in [5]. Here, the authors put together multiple narrowband detectors to maximize the throughput of secondary users limiting the interference with primary users. After that, several contributions appeared in the literature, for example, the one presented in [6]. In this work, the authors considered a genetic optimization in a collaborative multiband sensing scenario. The solution solved detection problems efficiently and increased the data rate. Another case appears in [7]; in this paper the authors consider an asynchronous dynamic spectrum access (DSA) algorithm in a wideband channel divided into several narrowband channels for finding the optimal sensing period time.

However, a modern point of view of multiband spectrum sensing applied to next-generation services is presented in [8]. In this contribution, most representative user detection techniques are classified. For the case of wavelet sensing, the authors mentioned this method provides a good analysis of the singularities of the spectrum. In the same way, a challenging system model is presented in [9]. The authors considered a hardware limitation for SUs in multiband spectrum sensing. Also, according to the traffic state, a proposed algorithm selects the best spectrum sensing strategy. An improvement of this work appears in [10]; here, channel correlation is considered in order to adapt the proposal to practical scenarios as broadcast television. In [11], a probabilistic access method in multiband spectrum sensing is presented. The authors classify spectrum occupancy as *likely*. Their results show how this classification improves data rates compared with classical spectrum sensing methods.

Spectrum detection in CR should be performed with high precision in order to avoid interfering with PUs. In the case of the Internet of Things (IoT) where many devices are transmitting, it is important to detect available spectrum spaces in order to maintain good network performance. For example, in [12] the authors present a solution to minimize the number of channels that have to be sensed by IoT nodes but keeping detection probabilities requirements. Results show an important reduction in energy consumption compared with similar spectrum sensing contributions.

**Figure 1:**
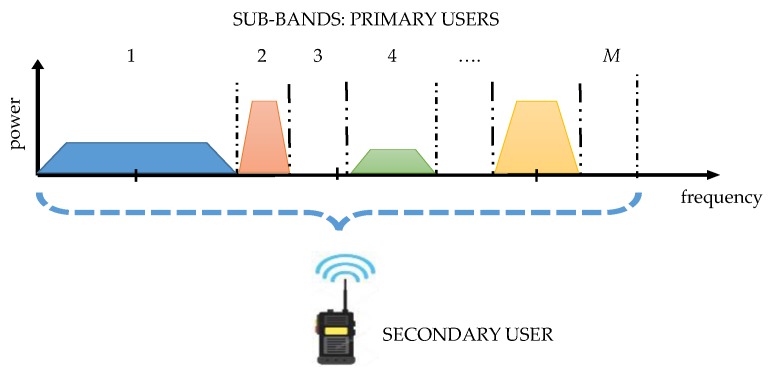
The multiband spectrum sensing concept in cognitive radio (CR) presented in [8].

The proposed methodologies in this work, for the multiband spectrum sensing stage, allow the detection of whether multiple bands (or channels) are occupied or not by PUs, and indicating perfectly the frequencies in which an SU could be placed, like is shown in Figure 1. For this, a large band spectrum containing *M* sub-bands with possible PU transmissions is considered and a combination of the continuous wavelet transform (CWT) [13], the multiresolution analysis (MRA) [14], and the Higuchi fractal dimension (HFD) [15] is explored. In the first methodology, the modulus maxima of the CWT of the reconstructed spectrum from the approximation coefficients issued from the MRA at a certain level of decomposition is used to detect the frequencies (edges) where the PUs are placed, generating in this way dynamic windows where the decision rule will be applied. In the second methodology, only the approximation coefficients of the MRA are used for frequency edge detection. The decision rule, for both methodologies, is constructed from the application of the HFD over the same reconstructed signal from the MRA. The HFD permits the detection perfectly of the presence of a PU or its absence (only noise detection) even in low signal-to-noise ratios, as it is shown in results, stem from real and simulated signals. The behavior of the HFD is very well known when it is calculated on a noise-like signal, of which the value tends to 2. When the signal is more regular, like a sinusoid or a rectangular pulse, the HDF value tends to 1. In this way, it is possible to differentiate the noise from a transmission signal establishing a correct threshold in the decision rule.

Some works are related to the proposed methodologies, especially using the CWT through its modulus maxima [16,17,18,19], to obtain the edges delimiting the different sub-bands. However, all these works base their decision rule on the energy of the coefficients presenting poor performances in low signal-to-noise ratios (a well-known characteristic of energy detectors). Besides, the edge detection is also affected by the broadband noise in the transmission signal or the apparition of impulse noise (generating false edge detection). Another more recent work [20] includes the modulus maxima of the CWT for edge detection but also the MRA for the decision rule. This rule is based in an energy detector of the coefficients from the MRA tracking also the maxima but in the discrete domain.

In [21] is proposed a double-threshold cooperative spectrum detection algorithm based on the Sevcik fractal dimension (SFD). In this work, the main idea is to detect the presence of primary users by analyzing the spectrum based on different characteristics of the SFD between signals and noise. The simulations results show that the algorithm can achieve high detection performance with low SNR. However, this technique only considers a single band, i.e., no edge detection is necessary. Besides, the method is only applied on simulated signals.

This work is organized as follows. In Section 2 theoretical bases from CWT, MRA, and HFD are briefly presented. In Section 3 the proposed methodology is developed. Simulations, the application on real signals, and results in Section 4 are discussed. Finally, in Section 5, conclusions and future work are delivered.

## 2. Theoretical Bases

In this section, the necessary background associated with the techniques used in the proposed methodology is briefly presented.

### 2.1. Continuous Wavelet Transform

The continuous wavelet transform of a function (signal) f(x) is defined by [13]:

(1) $$CWT_{f}\left(s,\tau \right)=\int_{-\infty }^{\infty }f\left(x\right)\psi_{s,\tau }^{*}\left(x\right)dx$$

where * represents complex conjugate and the functions family $\psi_{s,\tau }\left(x\right)$ is generated from the translation and scaling of an initial wavelet function ψ(x), sometimes called wavelet mother, and defined by:

(2) $$\psi_{s,\tau }\left(x\right)=\frac{1}{\sqrt{s}}\psi \left(\frac{x-\tau }{s}\right)$$

where *s* and *τ* are the scaling and translation factors, respectively. Only scale factors s>0 are used. Wavelets are dilated when the scale s>1 and they are contracted when s<1. In this way, an alternative to the time-frequency representations is given: the plane time (or translation)-scale. This representation, translation-scale, is more effective detecting singularities or discontinuities in the signal f(x). These transitions are located considering the modulus maxima of the CWT, defined by:

(3) $$\max\left[\left|CWT_{f}(s,t)\right|\right].$$

This mathematical tool is frequently used in many different domains, like image processing, control, biomedical, etc. In the specific case of spectrum sensing in CR, this technique is considered effective in detecting the transitions (edges) of the multiband spectrum [16,17,18,19,20].

### 2.2. Multiresolution Analysis

**Figure 2:**
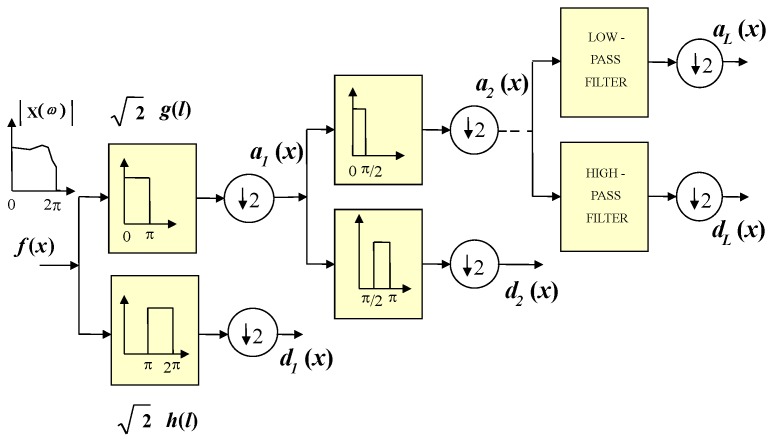
Implementation of the multiresolution analysis (MRA) based on a filter bank.

The multiresolution analysis was introduced by Mallat in 1989 [14]. This idea, involving embedded vector spaces, permits the decomposition (and reconstruction) of a signal f(x) using an orthonormal (or biorthogonal) basis. This complex idea has an easy interpretation through a bank of digital filters scheme, as it is shown in Figure 2. The decomposition equation is given by:

(4) $$\begin{array}{cc} f\left(x\right) & =approx_{L}\left(x\right)+\sum_{j=1}^{L}detail_{j}\left(x\right)\\ & =a_{L}\left(x\right)+\sum_{j=1}^{L}d_{j}\left(x\right) \end{array}$$

where $a_{L}\left(x\right)$ are the approximation coefficients at level *L* and $d_{j}\left(x\right)$ are the detail coefficients going from levels 1 to *L*. In this way the signal is filtered in octaves (i.e., each band is divided by two iteratively). The approximation coefficients permit, in the reconstruction process, having a smooth version of the original signal f(x). Detail coefficients give to the reconstructed signal the fine features. Based on these properties, the MRA can be used in the SS stage to get the tendency of the multiband spectrum in the approximation coefficients eliminating, in the reconstruction process, the contribution of the detail coefficients (containing the added noise to the transmitted signal or simply the noise floor).

MRA is implemented using a fast algorithm called *à trous*. It is important to mention that the MRA and the CWT are linked through the detail coefficients. The latter corresponds to a discrete version of the CWT in specific sampling points: the dyadic scale.

### 2.3. Higuchi Fractal Dimension

In general, a fractal curve has the property that each part of the curve can be considered a scaled-down image of the whole [22]. The fractal dimension is an index describing the *regularity* of a time series (in general, an array of *N* points sequentially ordered). Higuchi [15] modified the method proposed in [23] to calculate the fractal dimension and applied it not only to the simulated data but also to the time series stem from natural phenomena with turbulent behavior, obtaining a stable value of the fractal dimension.

The Higuchi fractal dimension returns a value in the interval [1,2], where the maximum value means that the evaluated series does not have a similarity with itself (completely *irregular*). However, for the minimum value, the self-similarity in the evaluated series is large (e.g., a periodic signal). From simulations, the HFD for a signal similar to *white noise* will be a value that tends to 2. This important feature is the base of the proposed decision rule in this work to differentiate the PU transmission from the noise itself. Indeed, ignoring the effect of an additive Gaussian noise on a PU transmission, the classical wireless transmission has shapes that can be considered as regular: an NRZ-CDMA pulse has a shape involving a sinus cardinal or an OFDM symbol, in the reception, and has a form of a pulse with small changes in its bandwidth. In this way, applying the HFD to this kind of shape in general will have a value near to 1, permitting an SU to be able to clearly differentiate the noise and a PU transmission. This premise is verified with detailed simulations presented in Section 4.

The method proposed by Higuchi [15] considers a time series of *N* points (or an ordered *N*-points sequence) x(1), x(2), x(3), …, x(N). From this series is constructed a new sequence $x_{k}^{m}$ fined by:

(5) $$x_{k}^{m}=\left\{x\left(m\right),x\left(m+k\right),\;x\left(m+2k\right),\ldots ,x\left(m+\left[\frac{N-m}{k}\right]k\right)\right\}\;m=1,2,\ldots ,k$$

where [] denotes the integer part, and both *k* and *m* are integers indicating the initial time and the interval time, respectively. The slope of the curve $L_{m}\left(k\right)$ in Equation (5), on a logarithmic scale, corresponds to the HFD.

(6) $$L_{m}\left(k\right)=\left\{\frac{N-1}{\left[\frac{N-m}{k}\right]\;k}\left(\sum_{i=1}^{\left[\frac{N-m}{k}\right]}\left|x\left(m+ik\right)-x\left(m+\left(i-1\right)k\right)\right|\right)\right\}/k$$

## 3. Methodologies

In this work, two methodologies to detect PUs in CRs were developed. The first methodology was based on the CWT to detect the edges in a multiband spectrum, creating in this way dynamic windows (i.e., windows of different lengths). In the second methodology, the edges detection stage via the CWT was replaced by directly using the approximation coefficients obtained from the MRA. In the next paragraphs, both methodologies are explained in detail.

### 3.1. First Methodology: MRA, CWT Modulus Maxima, and HFD

This methodology is described by the next steps:

Step 1. The received multiband spectrum by an SU, X(f), is decomposed via the MRA at defined level *L* giving the respective approximation and detail coefficients (see Equation (4)).

Step 2. From the obtained approximation coefficients, the spectrum is reconstructed eliminating in this way the broadband noise and only keeping the *tendency* (or smooth shape $X_{app}\left(f\right)$) of the multiband spectrum X(f).

Step 3. The CWT modulus maxima is applied on $X_{app}\left(f\right)$ to get the frequency edges and to conform dynamic windows for further analysis.

Step 4. Each conformed window is then processed to detect noise or a possible PU transmission. For this, the same approximation coefficients obtained in Step 1 are normalized and interpolated (to have the same samples as the dynamic windows). If the normalized approximation coefficients (NAC) are, on average, over a defined threshold of 0.7 then the values of the analyzed windows are probably a PU transmission. If NAC is lower than 0.7, it is practically sure that the transmission corresponds to noise.

Step 5. If NAC is lower than 0.7 then the Higuchi fractal dimension is applied directly on the analyzed section of the multiband spectrum X(f). In the other case, the HFD is applied on the reconstructed signal from Step 2 (i.e., $X_{app}\left(f\right)$).

Step 6. For each window, if the calculated HFD is lower than 1.85 (decision threshold), a PU transmission is detected. In another case, no PU is detected (only noise).

**Figure 3:**
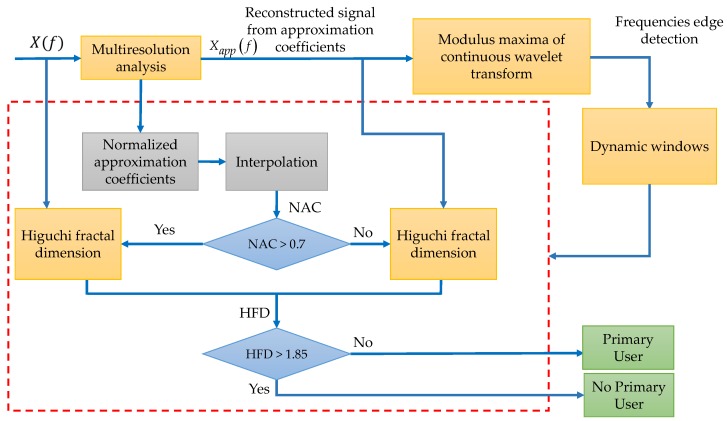
Complete scheme of the proposed first methodology. NAC: normalized approximation coefficients; HFD: Higuchi fractal dimension.

The complete scheme of this first methodology is shown in Figure 3.

Some important details, clarifying the proposed methodology, are discussed in the next paragraphs.

**Figure 4:**
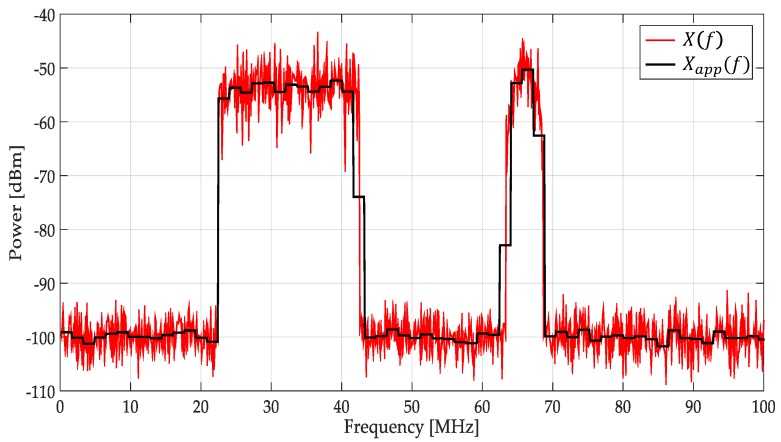
The result of applying MRA to X(f).

In Figure 4 is plotted a signal X(f) which represents a power spectral density in a wide frequency range. In this work, this signal was decomposed via the MRA using a Haar wavelet at level *L* = 4. The resulting smooth signal (denoted by $X_{app}\left(f\right)$ in Figure 4) was the reconstructed signal using only the MRA approximation coefficients (as is indicated in Step 1) showing notable changes in the presence of transmission by a PU and a clear *denoising* (Step 2). In this way, keeping only the MRA approximation coefficients permits, after reconstruction, the obtaining of the tendency of the signal.

**Figure 5:**
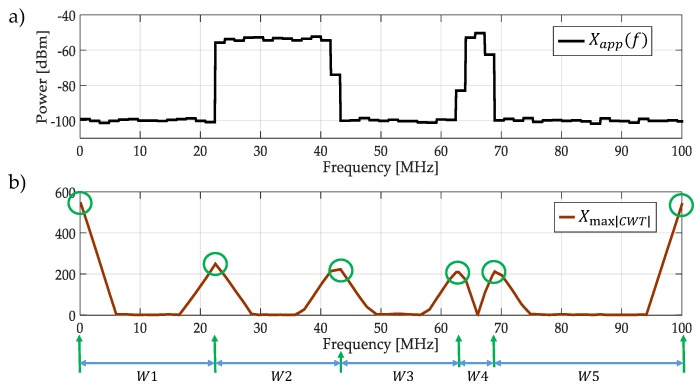
(**a**) $X_{app}\left(f\right)$ the approximation of X(f). (**b**) The result of applying the continuous wavelet transform (CWT) modulus maxima to $X_{app}\left(f\right)$ at scale 120 and its corresponding detected edges.

The result of applying the CWT modulus maxima to $X_{app}\left(f\right)$ is shown in Figure 5. In this figure (at the bottom) one scale of the CWT modulus maxima (scale 120) is only shown. Maxima of this scale are taken, and they correspond to sudden changes in frequencies of the smooth spectrum, as it is shown in Figure 5b in green circles. These frequency maxima values will be used to build *dynamic* windows (Step 3). These windows (five in this case), with the obtained frequency edges delimited by the green arrows in Figure 5b, feed the biggest block in the proposed algorithm in Figure 3.

**Figure 6:**
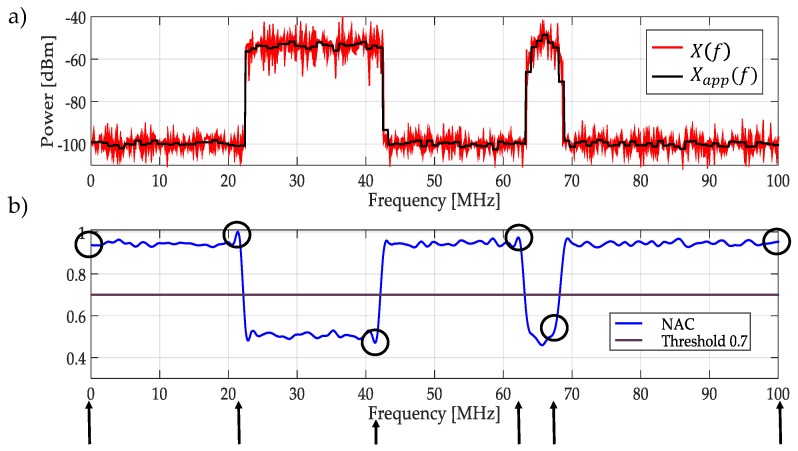
(**a**) The original and the approximated signal at decomposition level of 3. (**b**) The normalized and interpolated approximation coefficients (NAC) which mark the frequency edges and the same time that gives the dynamic windows and a threshold of 0.7.

**Figure 7:**
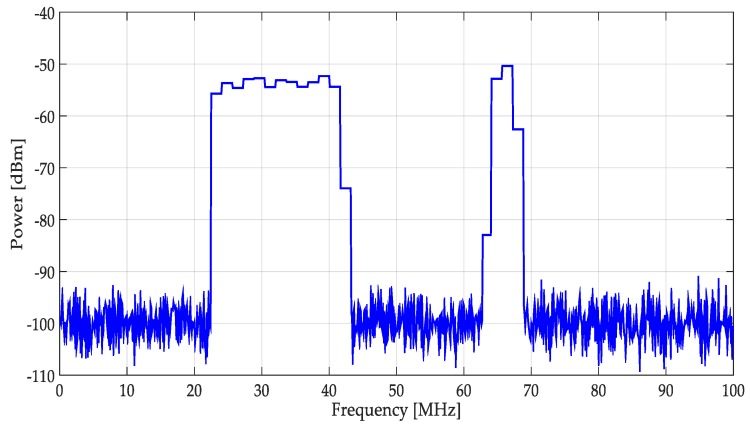
The resulting spectrum used in the decision rule to detect primary users (PUs).

Once the edges are detected, the original approximation coefficients from MRA are normalized and interpolated in order to have the same number of points aligned to the original signal X(f) (i.e., aligned with the obtained windows) as it is shown in Figure 6. If the average of the normalized approximation coefficients (NAC) aligned to each dynamic window is greater than 0.7, it means that in the frequency interval that is considered in the decision rule is directly the original signal X(f), else the decision rule will be applied to the spectrum $X_{app}\left(f\right)$ in the same interval (Steps 4 and 5). The value of 0.7 was set analyzing simulated signals. The result of the application of this threshold for this example, evidencing the existence of noise from the PU transmission, is shown in Figure 7.

As the final step, the resulting signal (spectrum shown in Figure 7) is used to determine a possible PU transmission. For this, the Higuchi fractal dimension is applied over each segment (window) of the spectrum (the NAC or the original signal). The decision threshold, to determine if there is a PU or not, is set to 1.85. If the HFD is greater than 1.85 that means, in this frequency interval, an SU could be placed or else a PU is transmitting. The threshold of 1.85 for the HFD was also set testing the methodology with simulated signals. It is important to point out that the choice to take directly or not the original signal involves the behavior of the HFD. Indeed, it is easier to decide about a PU transmission or noise, smoothing the spectrum (i.e., keeping the spectrum signal more *regular*) and keeping the *non-regularity* characteristic of the noise when the HFD is computed.

### 3.2. Second Methodology: MRA and HFD

**Figure 8:**
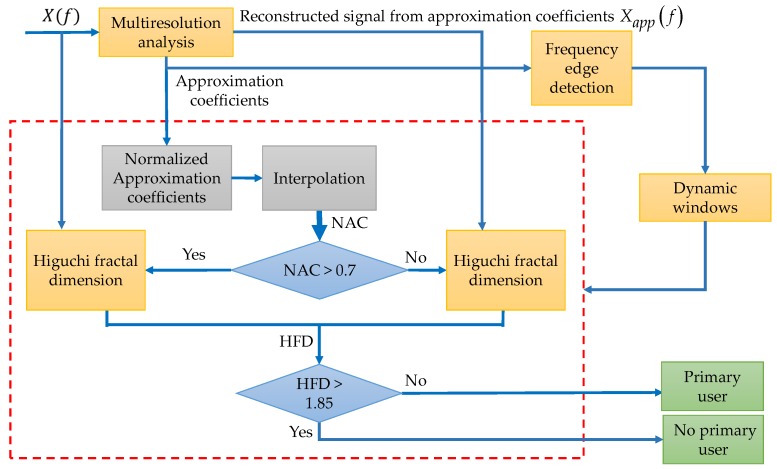
The second methodology described by a flow diagram.

Observing, in Figure 6, the behavior of the normalized and interpolated approximation coefficients obtained from MRA; a straightforward idea can be implemented: to use the changes presented along the frequency of these NAC to directly obtain the frequency edges in the multiband spectrum. In this way, the first methodology, explained before, can be modified yielding a second algorithm. This second proposal is described by the flow scheme presented in Figure 8.

Basically, Steps 2 and 3 of the first methodology are eliminated and substituted by considering the frequencies where the NAC changes based on the threshold of 0.7, i.e., the NAC going down to up from the 0.7 or vice versa. These changes will be the frequency edges necessary to construct the dynamic windows. In the case of the example, these detected critical points are marked in Figure 6 by black circles over the NAC.

Eliminating the CWT modulus maxima to detect the frequency edges gives a considerable diminution in the computational operations in the algorithm.

## 4. Implementations and Results

In this section, both proposed methodologies were implemented, and their performances evaluated. These methodologies were tested on simulated and real signals. Before presenting these implementations, another important issue was studied: the application of the Higuchi fractal dimension in the decision rule. For this, an analysis of its application to classical transmissions associated with the most representative wireless technologies in our days was developed.

### 4.1. HFD and Chosen Wavelet and Its Application on Wireless Transmission

**Figure 9:**
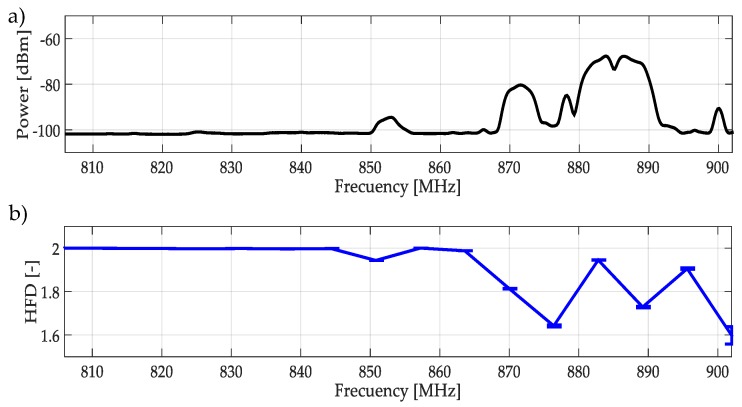
(**a**) The average of 300 multiband spectrum frames in the [806–902] MHz band. (**b**) The computed HFD (average) over these frames on uniform frequency windows.

The starting point for this work was thinking about applying the HFD to a multiband spectrum signal which is divided into uniform size windows, as shown in Figure 9. In Figure 9a, the average of 300 frames of real transmitted signals in the [806–902] MHz band, is plotted. In Figure 9b, the result of applying the HFD over frequency windows of the same size, is shown. Two important remarks can be pointed out:

In the multiple captured frames, the computed HFD of a signal without the presence of PU is a value that tends to 2.

The non-precise location of beginning and end of PU transmissions (due to the uniform frequency windows), introduces an HFD to be wrong, indicating a possible PU transmission as noise.

The second point is corrected, in both proposed methodologies, detecting the frequency edges and considering non-uniform size windows (dynamic windows). However, to set the threshold in the decision rule, it is necessary to be able to differentiate between noise (HFD ≈ 2) and a possible PU transmission. For this, HDF was applied on simulated signals representing classical access in wireless technologies (i.e., LTE, WIFI, 3G, etc.): OFDMA and CDMA. HFD was computed considering different SNR.

**Figure 10:**
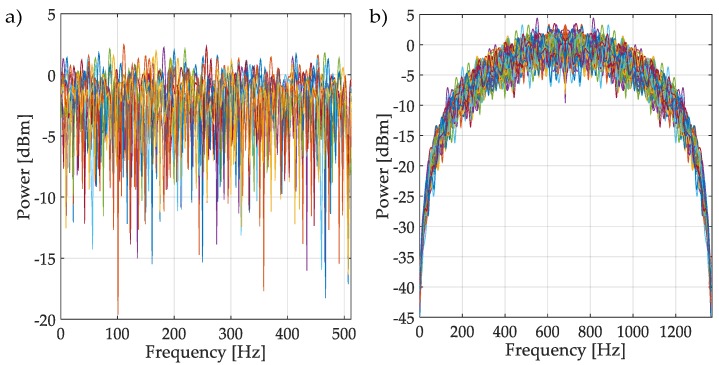
(**a**) Realizations of an OFDM spectrum. (**b**) Realizations of NRZ-CDMA spectrum.

**Figure 11:**
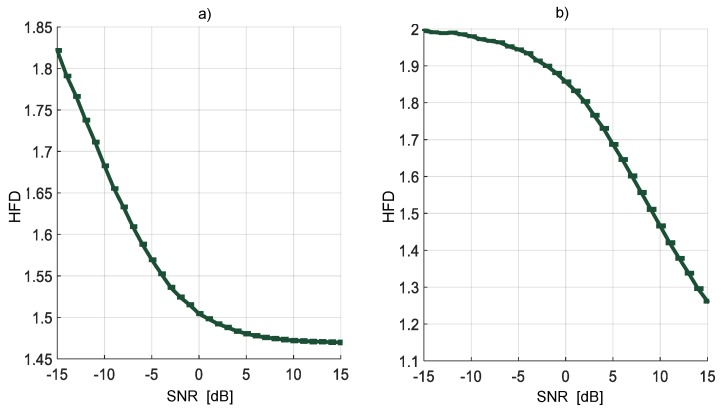
HFD of OFDM (**a**) and of NRZ-CDMA, (**b**) spectra with different SNR.

Multiple realizations of the synthesized OFDM and NRZ-CDMA symbols without noise are shown in Figure 10 with their respective mean and variance indicated in Table 1. The corresponding averaged HFD for these symbol realizations considering a wide interval of SNR, is displayed in Figure 11. From both Figure 10, Figure 11, and Table 1, we can conclude that the behavior of the computed HFD was around 2 where the symbols are practically noise (classical behavior of the HFD on white noise), however with high SNR, the values were not so different, being more difficult to differentiate which class of symbol was being transmitted. Nevertheless, this latter feature is not really important in spectrum sensing where the goal is to detect or not the presence of a PU. Another important thing to consider with these simulations is the sensibility of the HFD in presence of high levels of noise, giving wrong results (with values going to 2). Because of this, the direct application of the HFD is not feasible and it is then necessary to implement another processing. For this, in this work it was proposed to use the approximation coefficients from the MRA to get a smooth denoised spectrum, giving values of the HFD far from 2 and easily identifiable from the real noise.

**Figure 12:**
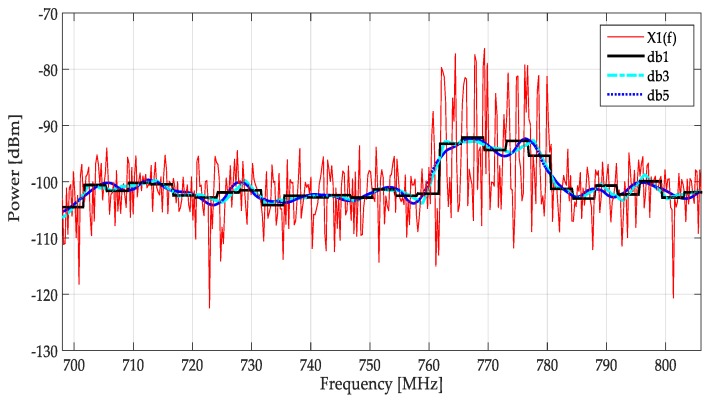
A real spectrum (in red) and the approximated signals from MRA using a Daubechies wavelet family.

The first consideration, to alleviate the above-mentioned problem, is to determine which kind of wavelet must be used. Figure 12 shows the different approximated signals from the MRA with different wavelet functions (Daubechies or db) implemented over a real signal. Although the tendency of the spectrum must be tracked using any wavelet of this family, the changes of a PU transmission are clearer with the db1 (or Haar) wavelet, which could improve the precision in locating the frequency edges in a multiband spectrum.

### 4.2. Simulated Signals

**Figure 13:**
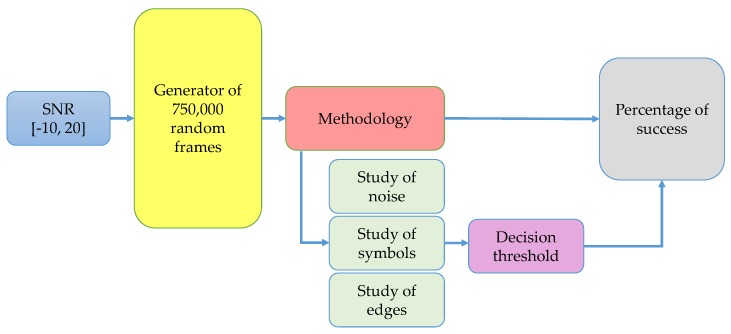
Scheme of the global simulation.

Both methodologies proposed in this paper were applied to a global simulation of 750,000 frames for each SNR value, varying in the interval [−10,20] dB and spaced by 2, as it is shown in Figure 13. In each frame, the number, type (OFDM or NRZ associated with CDMA), and position (frequency edges) of simulated symbols were generated randomly. Each frame consists of 1000 samples spaced by 0.1 MHz (i.e., an entire band of 100 MHz). Simulation parameters are summarized in Table 2. In Figure 13, three analysis blocks are added: studies of noise, symbols, and frequency edges. These studies serve to set the right threshold on the decision rule based on the HFD and the accuracy of detection frequency edges, as is detailed in the next paragraphs. Besides, the levels of decomposition of the MRA are 4 and 3 for first and second methodology, respectively.

#### 4.2.1. Results of First Methodology

**Figure 14:**
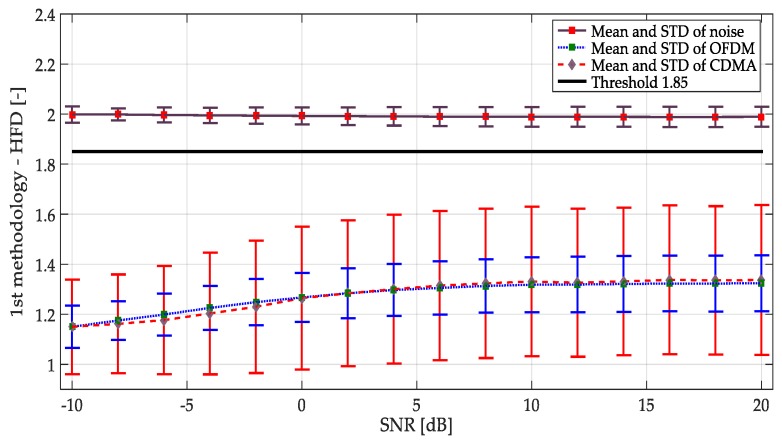
Mean and standard deviation (STD) for noise and simulated symbols (OFDM and NRZ-CDMA) applying the first methodology.

The result of symbols and noise studies when the first methodology was applied, is plotted in Figure 14. From here, it can be emphasized that the occurrence of an OFDM or NRZ-CDMA signal cannot be directly identified (i.e., the HFD mean is practically the same for both types of symbols at the same SNR). However, there was a clear division of what is a noise signal and what is a PU transmission. Based on these studies, for the first methodology, a threshold of HFD = 1.85 was chosen, as shown in Figure 14.

**Figure 15:**
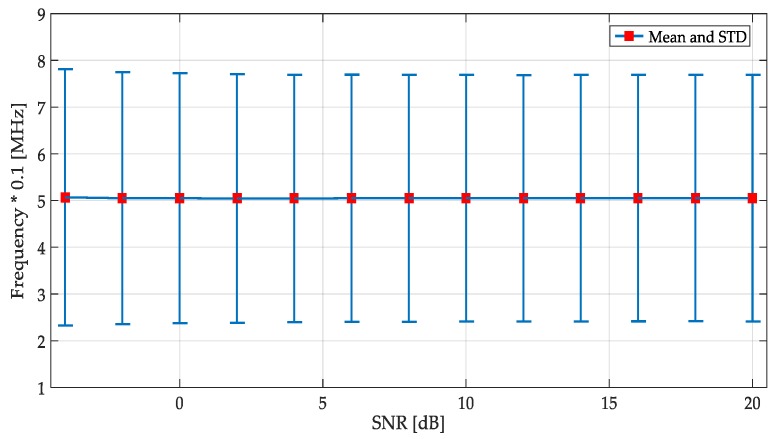
Study of frequency edge detection applying the first methodology.

The accuracy for the detection of the frequency edges (where a change of the simulated signal occurs) was also studied. These edges were the limits of the dynamic windows which for the first methodology were obtained with the CWT modulus maxima. Results from this study are shown in Figure 15. In these simulations, the performance of the proposed edges detector was excellent considering that the mean of the frequency deviation from the true simulated edge of the all detected values was five samples (around 5% from the true value). For SNR less than −4 dB (practically noise), edge detection was extremely imprecise, and it is not shown in the figure.

**Figure 16:**
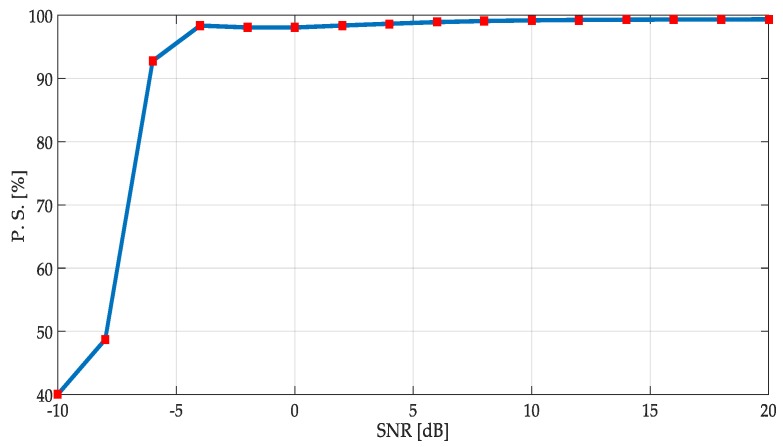
Percentage of success of the first methodology on simulated signals.

The global performance for each methodology was obtained considering the ratio of the total right transmission detections/true simulated values (PU transmissions) in percentage, i.e., the *percentage of success* of the global simulation. The percentage of success in applying the first methodology is shown in Figure 16.

Some important remarks can be pointed out from these results:

Performance of this proposed first methodology on an SNR above 0 dB was excellent. Indeed, the obtained percentage of success was over 95% of right detection, even more.

From values of SNR between 0 and −4 dB, the same performance was kept. If we consider these SNR values corresponding to a signal embedded in noise, the performances of the proposed technique were still excellent.

With SNR < −5 dB, the precision of the method was quickly degraded.

#### 4.2.2. Results of the Second Methodology

**Figure 17:**
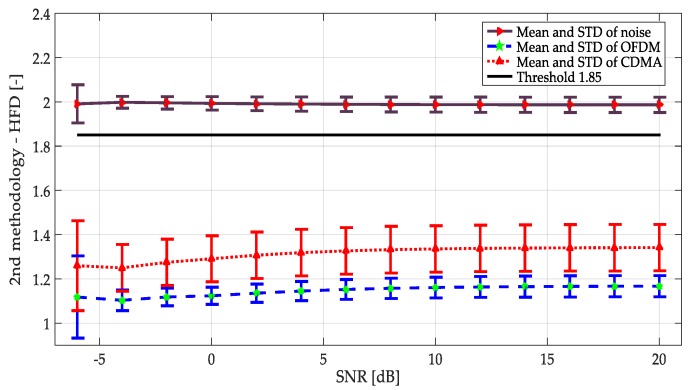
Mean and standard deviation for noise and simulated symbols (OFDM and NRZ-CDMA) applying the second methodology.

As the first technique, a study of symbols and noise was done implementing the second methodology (only using the approximation coefficients from MRA to detect the frequency edges and to establish the decision rule). Results are shown in Figure 17. The first difference regarding the first methodology was that the apparition of an OFDM signal or an NRZ-CDMA signal can be now identified. Indeed, the HFD mean values of these transmissions were clearly separated, even the variance levels were not really being touched; but this task was not the goal of this work. Secondly, there was a clearer division in the HFD between what was a noise signal from a PU transmission, permitting the establishment of a lower threshold for the HFD in the decision rule. However, this threshold was kept at 1.85, as in the case of the first methodology.

**Figure 18:**
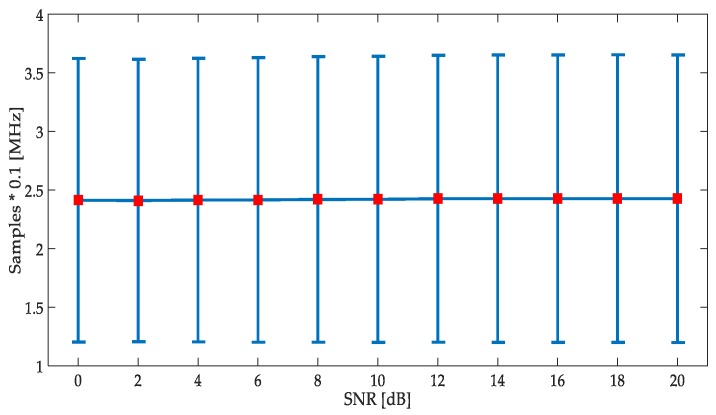
Study of frequency edge detection applying the second methodology.

A study of the accuracy in the frequency edge detection was done again. The results are displayed in Figure 18. These results show a better performance regarding the results obtained for the first methodology, diminishing the frequency deviation from the true simulated values to 2.4 samples (around 2.4% of true frequency deviation). The standard deviation was also diminished by 2. However, this edge detector was less precise (more susceptible to high noise levels) with very low SNR, in comparison with the detector based on the CWT modulus maxima.

**Figure 19:**
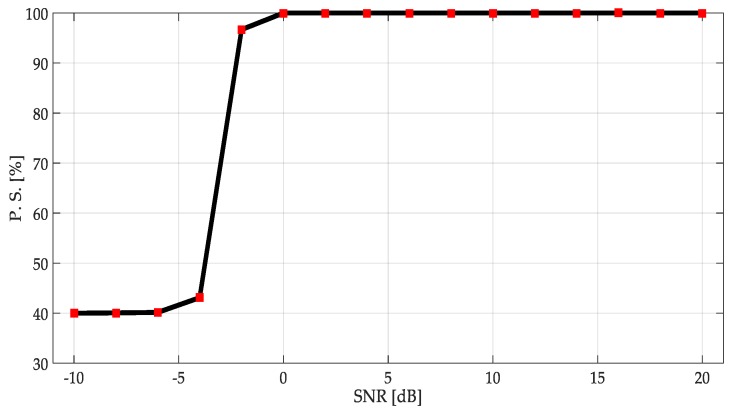
Percentage of success of the second methodology on simulated signals.

The percentage of success of applying this second methodology is plotted in Figure 19. In comparison with the first methodology, some important points can be remarked:

The second methodology on an SNR above 0 dB outperformed the first methodology. Indeed, the obtained percentage of success was practically around 100% of the right detection.

From the values of SNR between 0 and −4 dB, the first methodology had a better performance.

With SNR < −5 dB, the precision of both methods was quickly degraded.

### 4.3. Real Signals

To obtain real signals and characterize the multiband spectrum, a small spectrum occupancy measurement campaign was carried out. The data were obtained monitoring the frequency interval of [0.6–2.6] GHz during a complete week. An N9343C spectrum analyzer, obtaining 461 samples by frame of 100 ms each, was used. Data were collected using a directional HyperLOG antenna with a gain of 5 dBi.

**Figure 20:**
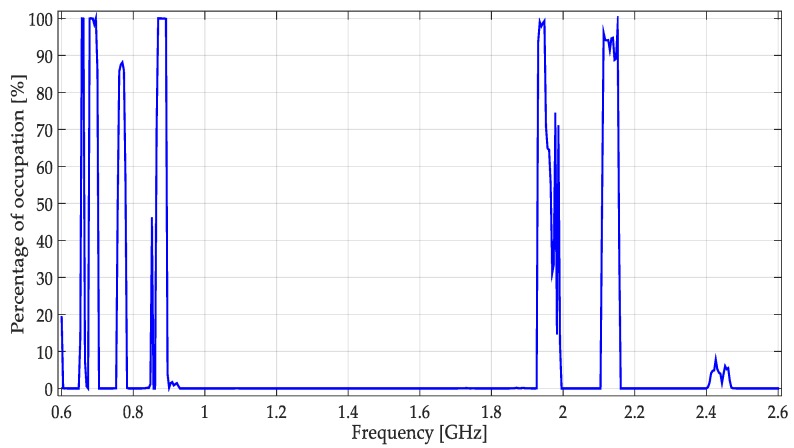
Percentage of occupation for a week in the frequency interval [0.6–2.6] GHz.

Results of the spectrum occupancy, indicated in percentage of occupation, are shown in Figure 20. It highlights the variability of the use of each band with time. From these results, six specific bands, being good candidates to implement the proposed multiband spectrum sensing techniques, were monitored again. The six specific monitored bands are indicated in Table 3. Each specific band was monitored during a complete day.

**Figure 21:**
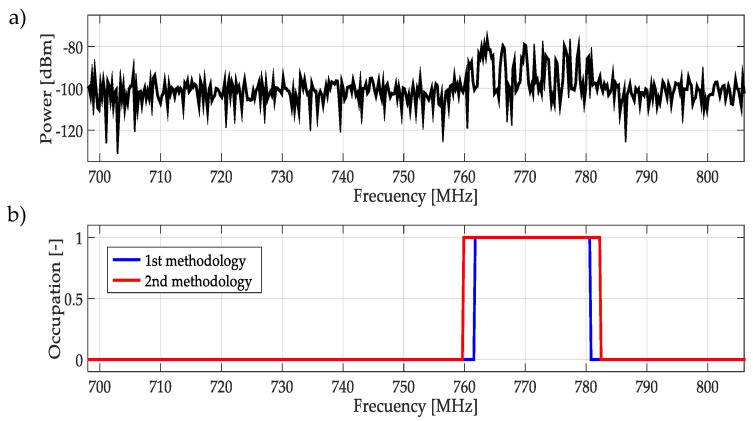
(**a**) A real signal obtained from the frequency band [698–806] MHz. (**b**) The result of applying both methodologies to the real signal in terms of spectrum occupancy. In this case, the second methodology gives a better frequency edge detection.

**Figure 22:**
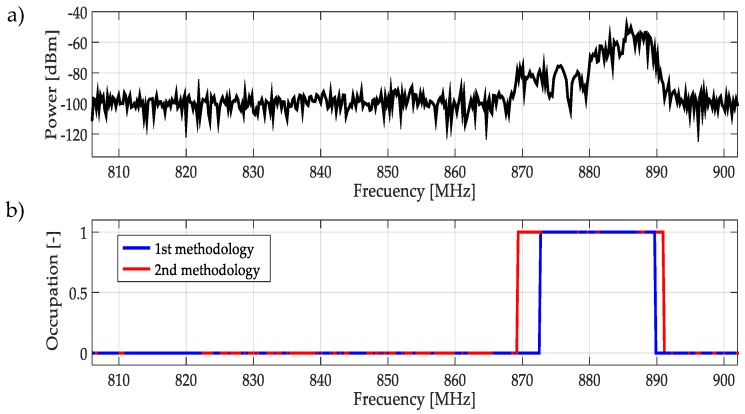
(**a**) A real signal obtained from the frequency band [806–902] MHz. (**b**) The result of applying both methodologies to the real signal in terms of spectrum occupancy. In this case, the second methodology gives a better frequency edge detection.

**Figure 23:**
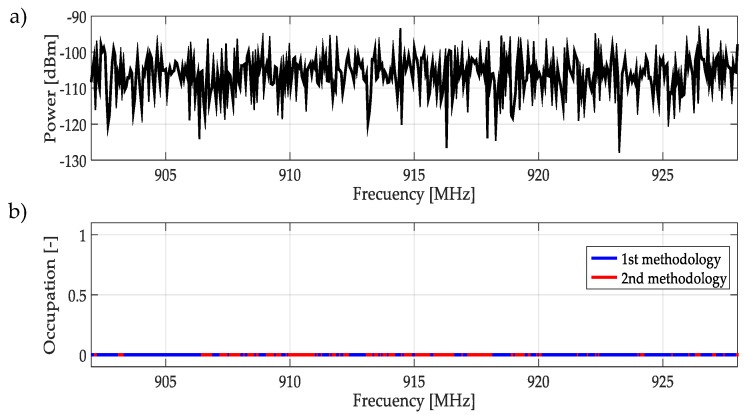
(**a**) A real signal obtained from the frequency band [902–928] MHz. (**b**) The result of applying both methodologies to the real signal in terms of spectrum occupancy. In this case, both methodologies do not correctly detect a PU.

**Figure 24:**
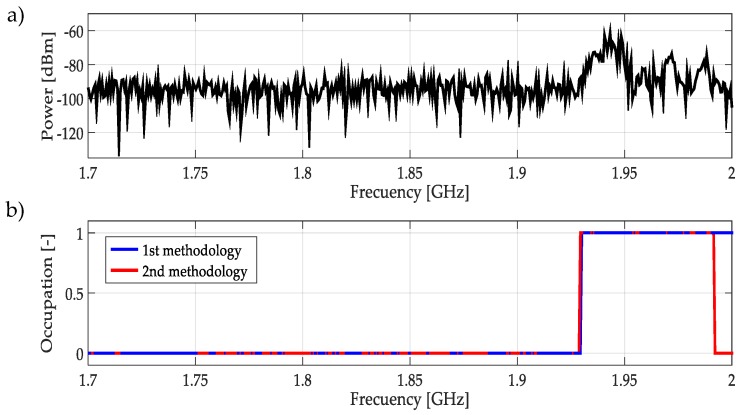
(**a**) A real signal obtained from the frequency band [1.7–2] GHz. (**b**) The result of applying both methodologies to the real signal in terms of spectrum occupancy. In this case, the second methodology gives a better frequency edge detection.

**Figure 25:**
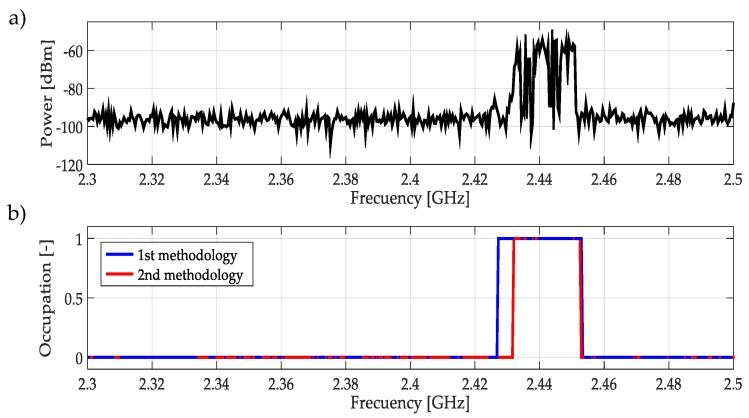
(**a**) A real signal obtained from the frequency band [2.3–2.5] GHz. (**b**) The result of applying both methodologies to the real signal in terms of spectrum occupancy. In this case, the first methodology gives a better frequency edge detection.

**Figure 26:**
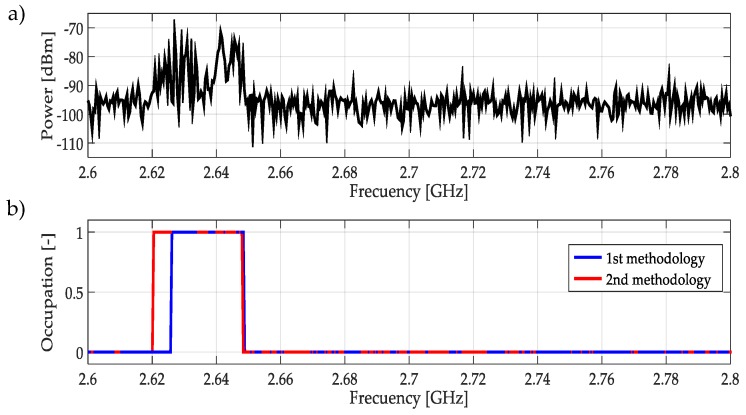
(**a**) A real signal obtained from the frequency band [2.6–2.8] GHz. (**b**) The result of applying both methodologies to the real signal in terms of spectrum occupancy. In this case, the second methodology gives a better frequency edge detection.

For real signals taken directly from the environment, it was not possible to control the PU transmission; unknowing its power and the exact frequency edges where it was transmitting. However, by inspection, most of the time it was to possible recognize one or more PUs in a specific multiband spectrum. Based on these facts, both methodologies were applied to data obtained in the different bands mentioned in Table 3. In this work, a challenging case (signal) for each band was chosen and results are plotted in Figure 21, Figure 22, Figure 23, Figure 24, Figure 25 and Figure 26. In these figures, a detected PU was indicated by a binary signal (placed on detected frequency edges): 0 for no PU transmission and 1 for a PU transmission.

Performances in general of both methodologies were remarkable. Sometimes edge frequency detection was better for the first methodology, as in the case of signals shown in Figure 25, but in general the second methodology outperformed slightly compared to the first. However, in all cases, PU transmission was perfectly detected. In the case of only noise, both methodologies worked perfectly, as shown in Figure 23. The great advantage of the second methodology was a lower computational complexity in comparison with the first methodology. Indeed, eliminating the CWT modulus maxima involving many computational operations gives a real opportunity to the second methodology to be considered in a real-time implementation, taking into account that the MRA uses a fast algorithm in its implementation.

**Figure 27:**
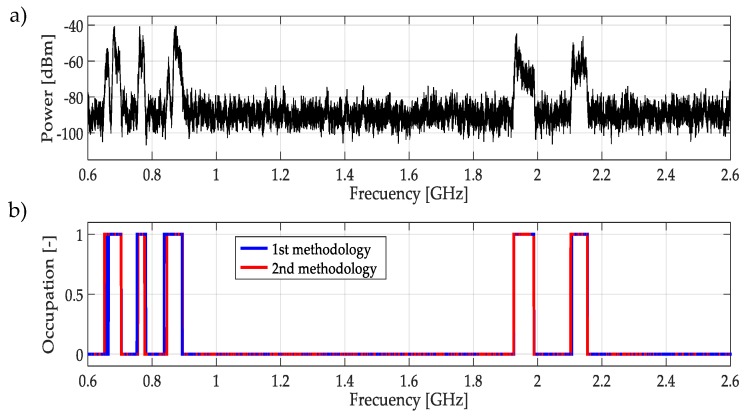
(**a**) A real signal obtained from the entire wideband of [0.6–2.6] GHz. (**b**) The result of applying both methodologies to the real signal in terms of spectrum occupancy. In this case, both methodologies have the same good performance.

Finally, both methodologies were applied to a real signal covering the complete frequency interval [0.6–2.6] GHz as shown in Figure 27. Both methodologies have correct detection in the face of PU transmission.

## 5. Conclusions

In this work, two novel methodologies based on wavelets and the Higuchi fractal dimension applied to multiband spectrum sensing were proposed. The difference between these two methodologies resides on the frequency edge detection. The first one used the CWT modulus maxima of the reconstructed signal from the MRA approximation coefficients, the second one used directly these coefficients, interpolating and normalizing them. The decision rule for both methodologies was based on the Higuchi fractal dimension. Some important conclusions can be delivered from the different results obtained from simulations and real signals:

The HFD of a series that corresponds to noise will have a value near to 2, since there is no self-similarity because the noise, in general, corresponds to a normal distribution.

Based on the first remark and from simulations, it was possible to discriminate noise from a PU transmission.

Better results were obtained considering the idea of windows of non-uniform size. Here, detection of frequency edges was very important. Both proposed methods for this task, the CWT modulus maxima or directly the approximation coefficient from MRA, give excellent results for edge detection; the second method having the advantage in a matter of implementation and computational complexity.

It can be concluded from the simulation results that the first methodology for multiband spectrum sensing worked for at least 95% of the cases with an SNR greater than or equal to 0 dB. This performance was improved with the second methodology reaching percentages of success around 98%, on average.

For real signals, it must be emphasized that the two proposed methodologies, applied to the bands mentioned in Table 3, have obtained favorable results in all the analyzed cases.

Finally, the possibility of a real-time implementation, especially of the second methodology, is feasible. As part of future work, including automatic learning techniques will be considered in order to avoid decision thresholds and to automate the full detection process. The methods presented here are thought to be implemented in a scenario for recent wireless communications. Also, the proposed methodologies will be implemented in a communications platform in order to know the computational complexity.

## Figures and Tables

**Table 1:** Mean and variance of the HFD applied to multiple simulated OFDM and NRZ-CDMA symbols without noise.

Symbol	HFD mean	HFD Variance
OFDM	1.476	0.001
NRZ-CDMA	1.0383	0.0001

**Table 2:** Simulation parameters.

Parameters	1st Methodology	2nd Methodology
Software	MATLAB 2014b	
SNR values	−10 to 20 dB spaced by 2	
Number of frames per each SNR value	750,000	
Number of symbols per frame	Random between [0,2]	
Bandwidth	100 MHz	102.4 MHz
Samples per frame	1000	1024
MRA decomposition level	4	3
Wavelet	Haar	

**Table 3:** Monitored frequency bands.

Frequency Band	Type of Communication
[698–806] MHz	Mobile and landline.
[806–902] MHz	Mobile and aeronautical mobile.
[902–928] MHz	Radiolocation, amateur, mobile, and fixed.
[1.7–2] GHz	Mobile and fixed.
[2.3–2.5] GHz	Radiolocation, amateur, mobile, and fixed.
[2.6–2.8] GHz	Aerial vehicles, radiolocation, and radio navigation.

## Abbreviations

CWT	Continuous wavelet transform
MRA	Multiresolution analysis
CR	Cognitive radio
SU	Secondary users
PU	Primary users
SS	Spectrum sensing
HFD	Higuchi fractal dimension
SFD	Sevcik fractal dimension
NAC	Normalized interpolated approximation coefficients
